# Platform, modality, and gamification effects on digit span task performance

**DOI:** 10.1016/j.isci.2026.116018

**Published:** 2026-05-20

**Authors:** Soner Türüdü, Ezgi Büşra Aktaş, Mehmet Can, Mehmet Kadir Ercan

**Affiliations:** 1 Department of Otorhinolaryngology/Head and Neck Surgery, University Medical Center Groningen, University of Groningen, Groningen, the Netherlands; 2 Research School of Behavioral and Cognitive Neuroscience, University of Groningen, Groningen, the Netherlands; 3 Department of Audiology and Speech Disorders, Institute of Health Sciences, Marmara University, İstanbul, Türkiye; 4 Department of Audiometry, Vocational School of Health Services, Erciyes University, Kayseri, Türkiye; 5 Department of Audiometry, Vocational School of Health Services, Karamanoğlu Mehmetbey University, Karaman, Türkiye; 6 Department of Otorhinolaryngology/Head and Neck Surgery, Radboud University Medical Center, Radboud University, Nijmegen, the Netherlands; 7 Donders Institute for Brain, Cognition and Behaviour, Radboud University, Nijmegen, the Netherlands

**Keywords:** Cognitive neuroscience, Human-computer interaction, Psychology

## Abstract

Web-based cognitive assessment raises questions regarding the psychometric equivalence of unsupervised online tests with traditional onsite administration. Gamification is often proposed to increase participant motivation, but its cognitive mechanisms remain debated. This study investigated the interactive effects of platform (onsite, standard online, and gamified online), stimulus modality (auditory and visual), and task direction (forward and backward) on digit span test performance using a within-subject design (*N* = 32). Standard online and onsite scores did not differ, supporting the validity of remote testing, and a robust auditory superiority effect emerged across platforms. The gamified platform produced higher scores than both standard versions, but this boost was not larger for the executive-demanding backward task, suggesting a general performance enhancement rather than selective modulation of the central executive. Both online platforms demonstrated good-to-excellent test-retest reliability without practice effects, supporting standardized online digit span tasks as psychometrically sound alternatives for cognitive assessment.

## Highlights

• Standard online DST performance is comparable to onsite performance
• Gamified online DST yielded higher scores than both standard versions
• Gamification boost was general, not selective for executive-load tasks
• Online DST platforms showed high reliability and no practice effects

## Graphical abstract

**Figure:**
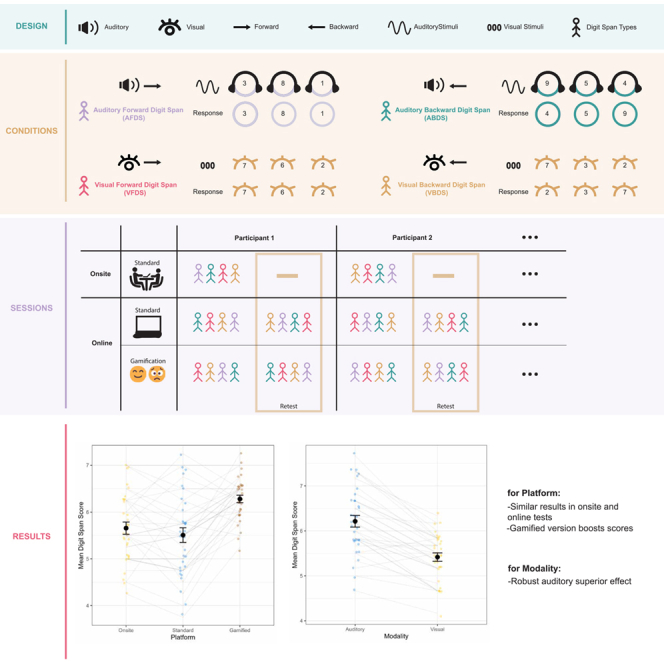

## Introduction

Working memory (WM) is defined as the limited-capacity system responsible for retaining and processing information, typically holding three to five meaningful items in young adults.^1^ This combination of storage and manipulation is fundamental for higher-level cognitive processes, such as reasoning and language.^2^ Because of the ability to integrate these two functions, WM is recognized as one of the three core components of executive functions (EFs), alongside inhibitory control and cognitive flexibility.^3^^,^^4^^,^^5^ Furthermore, since WM consists of different subcomponents, understanding their individual contributions is essential for the development and validation of effective assessment tools.^6^

The digit span test (DST) is one of the commonly used cognitive tools to assess attention, short-term memory, and WM capacity.^7^ The DST is typically administered in two directions as digit span forward (DSF) and digit span backward (DSB). The DSF task primarily measures the storage capacity of the phonological loop (PL), and during the task, participants are required to repeat digits in the same order as presented.^8^ Conversely, the DSB task is known as more demanding, and it requires individuals to recall the sequence in reverse order, which engages the central executive (CE) for active manipulation of information.^8^^,^^9^ This extra load is reported in a study by GrÉGoire and Van Der Linden that performance on DSB is typically about 1.5 digits lower than on DSF.^10^

The modality in which digits are presented is another important factor influencing DST performance. Auditory presentation often provides an advantage, as auditory stimuli gain direct and automatic access to the PL, which is a phenomenon known as the auditory superiority effect.^1^^,^^11^ On the other hand, visually presented digits must be subvocally recoded into a phonological format before they can be processed, which imposes an additional cognitive load.^6^

Even though the DST is widely used, there are some problems with the scoring. The major concern is combining DSF and DSB scores into a single composite measure, which has been shown to lack reliability. Many researchers advocate for analyzing the two subtasks (forward vs. backward) separately.^12^^,^^13^ Furthermore, literature shows inconsistent findings, with evidence suggesting observed impairments depend on the specific complexity and modality of the task.^14^ Tasks that place greater demands on the CE, such as DSB, seem to be more sensitive to external factors, including motivation and the testing environment.^9^ Such sensitivity is theorized to arise from the CE’s limited resources, which can be taxed by concurrent cognitive demands (e.g., recoding) and suboptimal states (e.g., low motivation). However, it remains unclear how these CE limitations interact with the uncontrolled nature of remote testing environments.

With the advances in digital technology, the influence of the testing environment has become a conspicuous research area. Digital cognitive assessments offer some advantages, such as standardized administration, increased efficiency, and the reduction of examiner-related variability.^15^^,^^16^ The computerized adaptations of the DST (e.g., DigiSpan) employ features like pseudo-randomization to minimize practice effects and enhance measurement accuracy.^15^^,^^17^ Nonetheless, the shift to unsupervised online testing has raised concerns regarding the comparability of onsite and online scores.^18^^,^^19^^,^^20^ An important theoretical limitation in current tele-assessment research is the inability to distinguish whether performance drops are due to technical differences or simply due to low participant engagement in monotonous tasks.^21^ Consequently, the literature has not yet reached a consensus on the comparability and reliability of onsite and online testing data.

A further challenge in both traditional and online cognitive testing is participant motivation. The repetitive and monotonous nature of many tasks frequently leads to reduced motivation, diminished effort, and compromised data quality.^22^ Gamification, which adds game elements to non-game tasks, has been proposed as a potential solution.^23^ According to self-determination theory (SDT), gamification can satisfy basic psychological needs, such as competence, thereby enhancing intrinsic motivation.^24^^,^^25^ However, the benefits of gamification are not guaranteed. Poorly designed gamified tasks may inadvertently increase cognitive load or introduce distracting elements, which is referred to as the “gamification paradox.”^22^^,^^26^^,^^27^ In particular, purely decorative or poorly integrated elements may act as seductive details, which increase extra cognitive load and compete for the same CE resources needed for the task itself. This generates a validity dilemma regarding whether gamification restores true performance by fixing motivation, or distorts the construct by overloading EFs. Despite the fact that this question is particularly important for high-load tasks like the DSB, few studies have systematically tested the interaction between gamification elements and specific executive load levels.

To address this gap, this study adopts an intermediate gamification approach^23^ to examine the interactive effects of three factors (platform, onsite, standard online, and gamified online; modality, auditory and visual; task direction, forward and backward) on DST performance. Unlike previous studies that treat gamification effects globally, we aim to test whether the gamification boost is preserved under high-executive load conditions (e.g., DSB) or whether the added cognitive cost of gamification diminishes its benefit. Specifically, we propose the on following hypotheses: (H1) performance the standard online DST will be comparable to onsite performance, whereas the gamified online platform will yield better scores than both standard versions; (H2) based on the auditory superiority effect, auditory presentation will produce higher digit span scores than visual presentation; (H3) the advantage of auditory presentation will be more pronounced for the DSB task than for the DSF task, as the visual-backward condition entails both recoding and manipulation costs; and (H4) the narrative gamified DST will lead to greater improvements in performance compared to the standard online DST, with the largest gains expected in backward tasks.

## Results

### Platform comparisons: Main effects and post-hoc analyses

Descriptive statistics for all tasks and platforms are presented in Table 1. Friedman tests indicated significant main effects of platform for visual forward digit span task (VFDST) (χ^2^[2] = 18.44, *p* < 0.001, *W* = 0.29), auditory backward digit span task (ABDST) (χ^2^[2] = 16.62, *p* < 0.001, *W* = 0.26), and visual backward digit span task (VBDST) (χ^2^[2] = 17.25, *p* < 0.001, *W* = 0.27). There was no significant difference for auditory forward digit span task (AFDST) (χ^2^[2] = 4.94, *p* = 0.085, *W* = 0.08).

**Table 1:** Descriptive statistics for digit span scores across all tasks and platforms

Platform by Task	AFDST Mean ± SD	VFDST Mean ± SD	ABDST Mean ± SD	VBDST Mean ± SD
Onsite	6.66 ± 1.00	5.84 ± 0.81	5.50 ± 0.92	4.62 ± 0.71
Standard online	6.31 ± 1.64	5.59 ± 1.21	5.53 ± 1.46	4.59 ± 0.88
Gamified online	6.97 ± 0.74	6.47 ± 0.57	6.31 ± 0.74	5.38 ± 0.71

AFDST = auditory forward digit span task, VFDST = visual forward digit span task, ABDST = auditory backward digit span task, VBDST = visual backward digit span task. All scores were shown as mean ± standard deviation.

**Figure 1:**
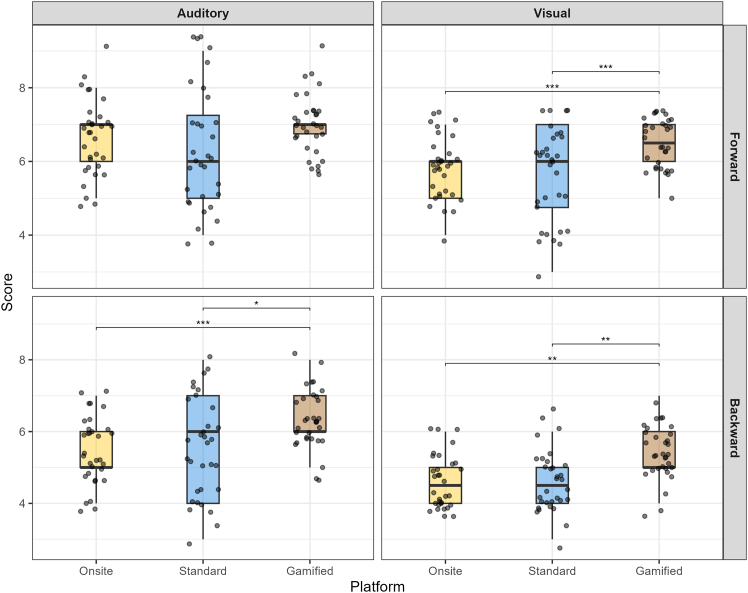
Performance scores across platforms, task directions, and modalities Plots illustrate digit span scores faceted by task direction (forward and backward) and stimulus modality (auditory and visual). Each set of figures shows data from the three platforms (onsite, yellow; standard online, blue; and gamified online, dark red). Boxplots represent the median and the interquartile range. Individual data points are overlaid. Horizontal brackets indicate significant pairwise comparisons. Friedman test with post-hoc Wilcoxon-signed rank test was used for pairwise comparisons. All *p* values were adjusted. ∗∗∗*p* < 0.001, ∗∗*p* < 0.01, ∗*p* < 0.05.

Post-hoc pairwise Wilcoxon signed-rank tests showed that gamified online scores were significantly higher than both onsite (VFDST *p* < 0.001, *r* = 0.700; ABDST *p* < 0.001, *r* = 0.731; VBDST *p* = 0.003, *r* = 0.607) and standard online scores (VFDST *p* < 0.001, *r* = 0.637; ABDST *p* = 0.013, *r* = 0.470; VBDST *p* = 0.003, *r* = 0.585). No significant difference was found for the AFDST gamified version compared to onsite (*p* = 0.163) or standard online (*p* = 0.167) versions. There was no significant difference between onsite and standard online scores for any task (AFDST *p* = 0.199, *r* = 0.204; VFDST *p* = 0.167, *r* = 0.225; ABDST *p* = 0.891, *r* = 0.046; VBDST *p* = 0.932, *r* = 0.077). Performance distributions and differences across tasks and platforms are visualized in Figure 1.

### Interaction effects: Modality and task direction

Analyses revealed that overall performance was significantly higher for auditory stimuli (6.21 ± 0.73) compared to visual stimuli (5.42 ± 0.53) (*V* = 487, *p* < 0.001, *r* = 0.836). Subsequently, we examined whether this modality advantage interacted with the platform. The results showed no significant difference in the auditory advantage score between the onsite and online platforms (*t*[31] = 0.08, *p* = 0.935, mean diff. = 0.016, 95% CI [-0.37, 0.40]).

**Figure 2:**
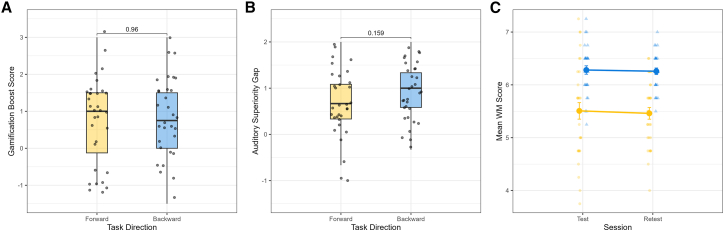
Interaction analyses and practice effects on WM performance (A) Gamification boost score compared across forward and backward task directions. Paired samples *t* test was used for statistical analysis. (B) Auditory superiority gap compared across task directions. Paired samples *t* test was used for statistical analysis. (C) Mean WM scores for standard (yellow line) and gamified (blue line) online platforms across the test and retest sessions. ART ANOVA with post-hoc Holm Bonferroni correction was used for statistical analysis. In (A) and (B), boxplots represent the median and interquartile range. Horizontal brackets indicate *p* values from paired *t* tests. In (C), points and lines represent the mean, with error bars indicating the standard error of the mean. Individual data points are overlaid in all figures.

To examine whether the benefit of gamification differed between forward and backward tasks online, a gamification boost score (mean gamified online score – mean standard online score) was calculated separately for each participant. A paired samples *t* test indicated that the gamification boost was not significantly different for backward tasks (mean 0.78 ± 1.15) compared to forward tasks (mean 0.76 ± 1.14) (*t*[31] = 0.06, *p* = 0.955, mean diff. = 0.02, 95% CI [−0.55, 0.58]). This interaction is visualized in Figure 2A.

Additionally, we examined whether the auditory superiority effect (auditory score > visual score) was larger for backward tasks than for forward tasks. A paired *t* test highlighted that the auditory superiority gap tended to be larger for backward (0.92 ± 0.61) than for forward tasks (0.68 ± 0.76); however, this difference was not significant (*t*[31] = 1.44, *p* = 0.159, mean diff. = 0.24, 95% CI [−0.10, 0.58]) (see Figure 2B).

### Longitudinal analysis: Practice effects

The ART ANOVA revealed a significant main effect of platform (*F*[1, 93] = 98.09, *p* < 0.001). Post-hoc Holm-Bonferroni-corrected contrasts confirmed that the gamified scores (test mean 6.28 ± 0.45; retest mean 6.26 ± 0.35) were significantly higher compared to standard online scores (test mean 5.51 ± 0.89; retest mean 5.46 ± 0.63) (*p* < 0.001). However, there was no significant main effect of session (*F*[1, 93] = 0.29, *p* = 0.593) or a platform and session interaction (*F*[1, 93] = 0.003, *p* = 0.957) (see Figure 2C).

### Psychometric reliability

The test-retest reliability analysis showed that all calculated ICC values were significant (all *p* < 0.001). Reliability coefficients were found to be excellent for ABDST (online ICC = 0.904). Reliability was good for AFDST (online ICC = 0.854; gamified ICC = 0.878), VFDST (online ICC = 0.846; gamified ICC = 0.804), and VBDST (online ICC = 0.877; gamified ICC = 0.834). Reliability was moderate for ABDST (gamified ICC = 0.748). The ICC coefficients and their 95% confidence intervals are presented in Table 2.

**Table 2:** Test-retest reliability of standard and gamified online digit span tasks

Platform (Online, *n* = 32)	Task	ICC	95% CI (lower, upper)	Reliability
Standard	AFDST	0.854	[0.724, 0.926]	good
Gamified	AFDST	0.878	[0.766, 0.938]	good
Standard	VFDST	0.846	[0.708, 0.922]	good
Gamified	VFDST	0.804	[0.636, 0.900]	good
Standard	ABDST	0.904	[0.815, 0.952]	excellent
Gamified	ABDST	0.748	[0.545, 0.868]	moderate
Standard	VBDST	0.877	[0.764, 0.938]	good
Gamified	VBDST	0.834	[0.686, 0.915]	good

ICC = intraclass correlation coefficient; CI = confidence interval; AFDST = auditory forward digit span task; VFDST = visual forward digit span task; ABDST = auditory backward digit span task; VBDST = visual backward digit span task; N = number of participants.

### Subjective survey analysis

Subjective outcomes from participants showed a positive trend. When asked for their overall preference (1 = prefer standard, 5 = prefer gamified), 18 of them indicated a preference for the gamified version (scores ≥4), while 11 reported no preference (score = 3), and three preferred the standard version (scores ≤2). On five-point Likert scales (where five indicates a high positive rating), participants reported moderate to high levels of overall enjoyment (mean 3.31 ± 0.82) and overall satisfaction (mean 3.91 ± 0.69) with the gamified experience. Participants perceived the gamification elements as having a positive effect on their motivation (mean 3.62 ± 0.71) and focus (mean 3.41 ± 0.80). The perceived task difficulty rating was favorable (Mean 3.69 ± 0.82). Given that 5 on the scale meant “definitely made it easier,” this indicates participants found the elements helpful rather than obstructive. Table 3 summarizes the descriptive statistics for the 10 subjective items.

**Table 3:** Descriptive statistics for subjective survey items

Item (five-point Likert Scale)	Mean ± SD	Median	Min	Max
Q1: Overall preference^a^	3.50 ± 0.72	4	2	5
Q2: Enjoyment	3.31 ± 0.82	3	2	5
Q3: Motivation effect	3.62 ± 0.71	4	2	5
Q4: Focus level	3.41 ± 0.80	3	2	5
Q5: Narrative effect	3.47 ± 0.51	3	3	4
Q6: Progress bar motivation	3.25 ± 0.88	3	1	5
Q7: Feedback usefulness	3.72 ± 0.89	4	1	5
Q8: Rank system motivation	3.28 ± 0.99	3	1	5
Q9: Perceived difficulty^b^	3.69 ± 0.82	4	2	5
Q10: Overall satisfaction	3.91 ± 0.69	4	3	5

Note: SD = standard deviation.

a Scale: 1 = definitely prefer standard, 5 = definitely prefer gamified.

b Scale: 1 = definitely made it harder, 5 = definitely made it easier.

Exploratory correlations between the gamification boost score (defined as the mean gamified score minus the mean standard score) and subjective survey variables were examined. After applying the Holm^28^ correction for multiple comparisons, no statistically significant correlations were found between the gamification boost score and any of the ten subjective variables (all *p* ≥ 0.128).

## Discussion

This study investigated the impact of platform (onsite, online), stimulus modality (auditory, visual), and gamification on WM performance, as measured by the DST. Our analyses yielded three key findings. First, in partial support of H1, performance on the standard online platform was comparable to that on the onsite platform, whereas the gamified online platform yielded superior performance. Second, as H2 predicted and consistent with the PL model,^1^ we observed a robust auditory superiority effect. Third, our interaction hypotheses were not supported. We did not find support for H3, which predicted that the auditory advantage would be larger for the backward span. Furthermore, while our minimalist gamification design worked (supporting the main effect predicted in H4), the performance boost was general, not disproportionately larger for the CE-loaded backward task. These results suggest that well-designed web-based assessments can be psychometrically robust and that gamification can improve performance, though perhaps through a general, rather than task-specific, mechanism.

A primary objective was to compare DST performance between onsite and online testing. Our results supported H1. We found no significant performance differences between the onsite and standard online platforms for any task variant. This finding aligns with literature demonstrating the validity of tele-assessments.^20^ Our data indicate that the potential environmental variability frequently seen in online research^21^ is mitigated by factors such as reduced performance anxiety^29^ or the elimination of examiner variability.^15^ Psychometrically, the online platforms were strong. Test-retest reliability (ICCs) was good to excellent for the standard tasks (ICCs 0.846–0.904). This indicates high performance consistency, crucially in the absence of any significant practice effects (see Figure 2C), a finding we attribute to the pseudo-randomization of stimuli.^21^

Nevertheless, we identified one notable exception. The reliability of the gamified ABDST (0.748) was significantly lower than that of its standard counterpart (0.904; see Table 2). This suggests that while gamification boosted mean performance, it may have also introduced inconsistent strategies upon retest, perhaps linked to the fluctuating nature of extrinsic motivation.^22^^,^^25^ For this most demanding task, it is plausible that the narrative and points inadvertently competed for limited CE resources, creating extra cognitive load.^30^ Such an added burden on an already saturated CE^6^ would lead to less stable performance. The test-retest reliability for the backward span was moderate to poor, as reported by Frois et al.,^31^ in contrast to the excellent reliability for the forward span.

In line with Baddeley’s^6^ WM theory and H2, we found a significant main effect of modality. The auditory superiority effect is presumed to reflect the direct access of spoken stimuli to the phonological store, whereas visual stimuli require resource-consuming subvocal recoding.^1^^,^^11^ This effect was robust across platforms, a finding which suggests the auditory superiority effect can be reliably captured in remote testing. Our H3, however, was not supported. We hypothesized that this auditory advantage would be magnified in the backward span task, arguing that the cognitive load from visual recoding would compound the executive load. While this was theoretically sound,^9^ we did not find statistical support. The mean auditory advantage was descriptively larger for backward tasks (0.92) than for forward tasks (0.68), but the difference did not reach statistical significance (see Figure 2B). Consequently, we lack the statistical evidence to conclude that the cognitive costs of visual recoding and active manipulation compound. Rather, our findings indicate that the auditory superiority effect remains relatively stable regardless of the additional executive load imposed by the backward task.

**Figure 3:**
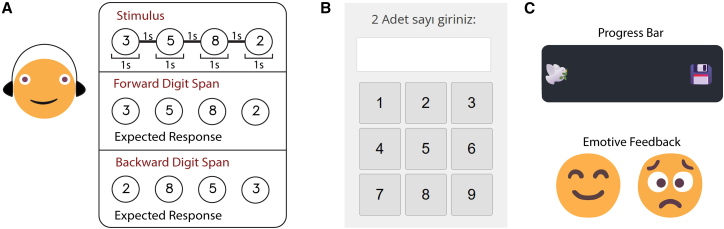
Experiment design, digit span tasks interface, and gamification elements (A) Shows an example of how a stimulus was presented, and ideal answers expected from participants in both forward and backward digit span tasks. (B) Demonstrates the interface of the response screen on which participants enter digits to give answers. (C) Illustrates smiley, frowning emojis, and a progress bar.

Consistent with H4, the game-up design in the present study was effective, causing higher scores than both standard versions (see Figure 1). As illustrated in Figure 3, the gamified version incorporated relatively minimal elements, including emotive feedback and a progress bar, while preserving the core digit span task structure. However, contrary to our expectation that gamification would selectively assist the executive-demanding backward tasks, the gamification boost was identical for both forward (mean 0.76) and backward (mean 0.78) tasks (see Figure 2A). Most importantly, this finding directly addresses the validity dilemma posed in the introduction. If our gamification elements had acted as seductive details,^32^ they would have competed for limited CE resources, disproportionately hampering performance on the DSB task. The absence of such a negative interaction suggests that our minimalist design successfully avoided the gamification paradox. Instead of creating construct-irrelevant variance by overloading the executive system, the gamification elements likely provided a general upregulation of arousal or attention, which benefits the PL (forward) and CE (backward) components equally. This improvement across both task directions suggests that gamification did not specifically offset the cognitive costs associated with active manipulation. Rather than selectively reducing the burden of the CE, the gamification elements appear to have fostered a platform-specific increase in sustained task engagement that supported both mere storage and active processing.

Intriguingly, the subjective survey data revealed a disconnect. Although objective performance improved, participants reported only moderate levels of enjoyment (3.31/5.00) and motivation (3.62/5.00; see Table 3), and we found no significant correlations between these ratings and the performance boost. This discrepancy illustrates the gamification paradox,^22^ which strongly implies that the performance boost was not mediated by self-reported positive affect,^33^ a finding that is inconsistent with some studies where positive affect was a key mediator for reducing task disengagement.^27^ Instead, this finding can be explained by SDT. The relatively high rating for feedback usefulness (3.72/5.00) suggests that the gamification elements (e.g., progress bar, emotive feedback, etc.) satisfied the basic need for competence. According to SDT, satisfying the need for competence can sustain goal-directed effort even if participants do not report increased enjoyment.^25^^,^^34^ Additionally, these elements may have helped by simply reducing monotony or maintaining alertness through attentional capture.^22^

The present study’s advantages include the direct testing of interaction effects, the psychometric evaluation, and the within-subjects design that compares three platforms. The absence of practice effects also strengthens our interpretation. Future research should aim to replicate these findings in large, diverse populations using a fully counter-balanced design and investigate the precise mechanisms of the gamification boost using objective measures (e.g., EEG).^27^ Another promising area is the investigation of various gamification designs and personalized approaches that are customized to user characteristics.^35^^,^^36^

### Limitations of the study

Several limitations must be noted. First, a potential order confound was introduced because the platform order was fixed (standard followed by gamified). However, data from the retest session argue against a simple practice effect. If the improvement were solely due to practice, standard scores in the second session should have remained elevated. Instead, they returned to baseline levels (see Figure 2C), and the performance boost only re-emerged with the gamified platform. Additionally, the risk of this confound is minimized by the test-retest results, which showed no significant platform and session interaction (*p* = 0.957). Second, the sample was predominantly composed of young adults, which restricted its generalizability. Third, the uncontrolled online environments introduce potential variability. Although task characteristics may discourage straightforward cheating, the potential for distractions or the use of aids cannot be entirely disregarded. Fourth, gender was not analyzed as a moderating variable since the sample size was not powered for between-group comparisons, which constrains the applicability of the findings with respect to potential gender-based differences. Finally, the findings are specific to the game-up design that we implemented in the study. Different elements (e.g., leaderboards, time pressure, etc.) could result in different cognitive effects.

In conclusion, the present study shows that standardized online digit span tasks are psychometrically sound and that our gamified version, in particular, improved overall performance to a level surpassing that of onsite testing. We demonstrate that gamification can enhance performance without selectively distorting EFs. The observed performance gain was general, not targeted. We found no evidence that it disproportionately enhanced the CE; rather, both storage and executive tasks were equally improved. These results support the use of standardized, engaging digital tools for cognitive assessment, which offer valuable insights for interdisciplinary researchers in psychology, digital health, and human-computer interaction regarding the complex mechanisms by which gamification influences performance.

## Resource availability

### Lead contact

Further information and requests for resources should be directed to and will be fulfilled by the lead contact, Mehmet Kadir Ercan (mehmetkadir.ercan@radboudumc.nl).

### Materials availability

This study did not generate new unique reagents.

### Data and code availability

• Behavioral data reported in this article will be shared by the lead contact upon request.
• This article does not report original code.
• Any additional information required to reanalyze the data reported in this article is available from the lead contact upon request.

## Acknowledgments

We thank all participants who took part in this study. We also thank Göksu Atmaca Ercan for her valuable contribution to preparing the graphical abstract. This work was supported by funding provided by the Turkish Ministry of National Education to S.T. and M.K.E.

## Author contributions

S.T.: conceptualization, funding acquisition, methodology, formal analysis, visualization, and writing – original draft; E.B.A.: conceptualization, methodology, data curation, and writing – original draft; M.C.: conceptualization and data curation; M.K.E.: conceptualization, funding acquisition, and writing – review and editing.

## Declaration of interests

The authors declare no competing interests.

## STAR★Methods

### Key resources table

**Table:** 

REAGENT or RESOURCE	SOURCE	IDENTIFIER
**Deposited data**		

Behavioral digit span data	This paper	Available from the lead contact upon request

**Software and algorithms**		

MATLAB R2023b	The MathWorks Inc.	RRID:SCR_001622
jsPsych	de Leeuw,^37^ 2015	RRID:SCR_023508
JATOS	Lange et al.,^38^ 2015	https://www.jatos.org/
R (version 4.4.3)	R Core Team,^39^ 2021	RRID:SCR_001905
G∗Power 3.1	Faul et al.,^40^ 2007	RRID:SCR_013726

**Other**		

Sennheiser HD 280 Pro headphones	Sennheiser	Cat# HD 280 PRO

### Experimental model and study participant details

#### Participants

Thirty-two native Turkish adults (mean age 25.2 years ± 5.44; age range: 18–41; self-reported gender: 19 women, 13 men) participated in the onsite and online testing. Participants were highly educated and reported having no neurological or cognitive impairment. We characterized normal hearing using standard clinical pure-tone audiometry. All had normal hearing thresholds of ≤20 dB hearing level (HL) bilaterally between 250 and 8000 Hz. Participants provided written informed consent. Information on ancestry, race, ethnicity, and socioeconomic status was not collected. The study protocol received approval from the Karaman University Faculty of Medicine Ethics Committee (09-2025/145) and was conducted in accordance with the Declaration of Helsinki.

### Method details

#### Procedure and study design

A within-subjects repeated-measures design was employed to compare working memory performance across three testing platforms: onsite, standard online, and gamified online.

In the onsite session, following the pure-tone audiometry, participants performed the Auditory Forward Digit Span Task (AFDST), Auditory Backward Digit Span Task (ABDST), Visual Forward Digit Span Task (VFDST), and Visual Backward Digit Span Task (VBDST). The DST procedures followed WAIS-III principles,^41^ where two trials were presented per sequence length, starting at 3 digits for forward and 2 digits for backward span. Sequence length increased by one if at least one trial was correct. Testing stopped after two consecutive failures at the same length. For each task, the span score was defined as the maximum sequence length a participant successfully recalled. A brief practice round, consisting of two trials with 2-digit sequences followed by one trial with a 3-digit sequence, was administered before each task.

After the onsite session, participants received unique links and instructions for completing the standard online and gamified online sessions at a place and time of their convenience. The order of four tasks (AFDST, ABDST, VFDST, VBDST) was randomized for each participant within each session. There was a minimum of one day break between standard and gamified online test sessions. Participants first completed the standard online session, followed by the gamified online session. Approximately two weeks later, participants repeated the online sessions with new, unique links. For auditory tasks, participants were instructed to use headphones. Before starting the test, they were first required to pass the Huggins pitch headphone screening test.^42^ After that, participants performed a calibration procedure, adjusting their device volume to a self-determined most comfortable level (MCL) using a sample digit stimulus (spoken digits 3-5-8 presented at one-second intervals).

#### Gamification elements

The standard online DST interface and procedure were augmented with specific gamification elements consistent with a game-up design approach,^23^ aiming to enhance engagement without fundamentally altering the core task mechanics or visual presentation significantly. We introduced five specific gamification elements: (1) a brief narrative theme (framing the task as an agent infiltrating a data center), (2) a visual progress bar (e.g., a moving icon representing progress through the task sequences), (3) performance-based ranks (e.g., security titles like ‘Analyst candidate’ awarded based on achieving certain span lengths), (4) points for correct trials based on sequence length, and (5) immediate emotive feedback (e.g., smiley/frowning faces; see Figure 3C). Crucially, the core task mechanics, such as stimulus presentation, trial structure (see Figure 3A), and response input (numeric keypad; see Figure 3B) remained identical to the standard online version.

#### Subjective measures

Upon completing the online session, participants were directed to a custom-developed JATOS (Just Another Tool for Online Studies) questionnaire. This instrument was designed to capture subjective evaluations of the gamified experience and to profile participants’ video gaming backgrounds (frequency, duration, platform preferences, and self-rated familiarity). The items first established an overall preference for the gamified or standard version. Subsequently, a series of 5-point Likert-type scales quantified constructs such as enjoyment, motivational impact, attentional focus, perceived task difficulty, and overall satisfaction. Additional items solicited targeted feedback on the perceived utility of the individual gamification components employed. The full questionnaire is available in English in Document S1.

#### Apparatus

Digit span tasks employed sequences of digits ranging from 1 to 9 for both visual and auditory modalities. The auditory task material was recorded by a native male speaker in a professional studio, with 44.1 kHz, 24-bit mono. Auditory stimuli were presented via calibrated headphones (Sennheiser HD 280 Pro) at 65 dB SPL. In visual span tasks, digits were presented as visual stimuli at the center of the screen. During the onsite testing, all participants were placed in a sound-treated room in front of a computer screen at a comfortable viewing distance (about 50 cm). Onsite testing was conducted using MATLAB (R2023b; The MathWorks Inc.) on a standard PC in a quiet laboratory. The jsPsych library^37^ was used to build both online tests (standard and gamified). The JATOS platform^38^ was then employed for their web-based administration. Participants used their own devices (desktops or laptops). As online sessions were unsupervised, variables related to the participants’ display hardware (e.g., screen size, resolution) and viewing distance were not controlled or recorded. A custom-designed onscreen numeric keypad facilitated responses via mouse click for both onsite and online testing.

### Quantification and statistical analysis

All statistical analyses were performed in R (version 4.4.3; R Core Team 2021)^39^ with a significance level of *p* < 0.05. Non-parametric tests were selected for platform comparisons because the residuals of the corresponding parametric models deviated from normal distributions (Shapiro-Wilk p < 0.05), with all multiple comparisons controlled for using the Holm-Bonferroni method. Since the onsite condition lacked a retest session, a fully crossed 3 (platform) by 2 (session) factorial model was not possible. Therefore, cross-sectional platform comparisons and longitudinal practice effects were modelled separately. Friedman tests assessed overall differences across the three platforms (onsite, standard online, and gamified online) for each task (AFDST, VFDST, ABDST, and VBDST), followed by pairwise Wilcoxon signed-rank tests with correction. To test for interaction effects, first, the difference in the auditory advantage score (mean auditory score – mean visual score) between onsite and standard online platforms was assessed using a paired-samples t-test. Second, the difference in gamification boost score (mean gamified score – mean standard score) between online forward and backward tasks was assessed using a paired-samples t-test. These parametric tests were deemed appropriate as the resulting difference scores were distributed normally (Shapiro-Wilk *p* > 0.05). A power analysis (G∗Power 3.1)^40^ indicated 30 participants would be required to detect a medium-sized effect for this interaction with 75% power (α = .05, two-tailed). Practice effects for the online platforms were assessed using a 2 (session: test, retest) by 2 (platform: standard and gamified online) Aligned Rank Transform (ART) ANOVA on the mean scores. Follow-up analyses were conducted using post-hoc contrasts with correction. The number of asterisks indicates different significance levels for statistical test results, which was defined as ∗∗∗: *p* < 0.001; ∗∗: *p* < 0.01; ∗: *p* < 0.05. Test-retest reliability for the standard and gamified online conditions was evaluated for each task score using Intraclass Correlation Coefficients (ICC, two-way mixed, absolute agreement).^43^ ICC values were interpreted following Koo and Li (2016).^44^ For the survey analysis, descriptive statistics were calculated. Exploratory correlation analyses were conducted to examine the relationship between a gamification boost and subjective variables.

## Supplemental information

Document S1. Subjective questionnaire (English version), related to STAR Methods

## Footnotes

Supplemental information can be found online at https://doi.org/10.1016/j.isci.2026.116018.
