# Supplementary material for: Platform, modality, and gamification effects on digit span task performance
Source: iScience. 2026 May 20;29(6):116018. doi: 10.1016/j.isci.2026.116018 (PMC13214330; doi:10.1016/j.isci.2026.116018)
Supplement: Document S1. Subjective questionnaire (English version), related to STAR Methods [file mmc1.pdf]

iScience, Volume *29*

Supplemental information

Platform, modality, and gamification effects on digit span task performance

Soner Türüdü, Ezgi Büşra Aktaş, Mehmet Can, and Mehmet Kadir Ercan

Data S1: Gamification feedback questionnaire

Introductory text presented to participants

This questionnaire aims to understand your thoughts on the gamified version of the memory task you recently completed. Please answer by considering both the standard and gamified versions.

Part 1: Demographic Information

1. Your Age:

    [Numeric input field]

2. Your Gender:

    ▢ Female

    ▢ Male

    ▢ Other

    ▢ Prefer not to say

Part 2: Game Experience and Gamification Familiarity

3. How often do you play video/computer games?

▢ Never

▢ Very Rarely (A few times a year)

▢ Rarely (A few times a month)

▢ Occasionally (1-3 days a week)

▢ Frequently (4-6 days a week)

▢ Every day

4. What is your daily game time?

▢ I don't play / Not applicable

▢ Less than 1 hour

▢ Between 1-2 hours

▢ Between 2-3 hours

▢ Between 3-4 hours

▢ More than 4 hours

5. Which game genres do you prefer? (You can select more than one)

▢ Action (Fighting, Adventure, Platformer, etc.)

▢ Strategy (RTS, TBS, etc.)

▢ Role-Playing Games (RPG, MMORPG, etc.)

▢ Simulation

▢ Sports / Racing

▢ Puzzle / Brain Teasers

▢ Shooter Games (FPS, TPS, etc.)

▢ MOBA (e.g., LoL, Dota)

▢ Casual Games

▢ Story-Driven / Interactive Fiction

▢ Other

▢ I don't play games / Not sure

6. On which platforms do you usually play? (You can select more than one)

▢ Computer (PC/Laptop)

▢ Game Console (PlayStation, Xbox, Switch, etc.)

▢ Mobile Device (Smartphone / Tablet)

▢ Handheld Console (3DS, Vita, etc. - excluding Switch)

▢ Other

▢ I don't play games / Not sure

7. How familiar are you with progression systems in games (e.g., leveling up, earning points, getting ranks)?

[5-point Likert scale]

▢ 1 = Not Familiar at all

▢ 2 = Slightly Familiar

▢ 3 = Moderately Familiar

▢ 4 = Considerably Familiar

▢ 5 = Very Familiar

Part 3: Subjective Experience Questionnaire (5-point Likert scale)

1. Overall, do you prefer the gamified version or the standard version?

 ▢ 1 = Definitely Prefer Standard Version

 ▢ 2 = Prefer Standard Version

 ▢ 3 = No Preference / Undecided

 ▢ 4 = Prefer Gamified Version

 ▢ 5 = Definitely Prefer Gamified Version

2. How enjoyable did you find the gamified version?

 ▢ 1 = Not Enjoyable at all

 ▢ 2 = Slightly Enjoyable

 ▢ 3 = Moderately Enjoyable

 ▢ 4 = Enjoyable

 ▢ 5 = Very Enjoyable

3. To what extent did the gamified version affect your motivation to continue the task?

 ▢ 1 = Decreased my motivation

 ▢ 2 = Slightly decreased my motivation

 ▢ 3 = No effect

 ▢ 4 = Slightly increased my motivation

 ▢ 5 = Greatly increased my motivation

4. How focused/immersed did you feel during the gamified version?

 ▢ 1 = Not Focused at all

 ▢ 2 = Slightly Focused

 ▢ 3 = Moderately Focused

 ▢ 4 = Considerably Focused

 ▢ 5 = Completely Focused

5. To what extent do you think the agent, pigeon, and data center-themed narrative affected your engagement with the task?

 ▢ 1 = Very Negatively Affected

 ▢ 2 = Negatively Affected

 ▢ 3 = No Effect

 ▢ 4 = Positively Affected

 ▢ 5 = Very Positively Affected

6. How motivating did you find the pigeon moving towards the data center (progress indicator)?

▢ 1 = Not Motivating at all

▢ 2 = Slightly Motivating

▢ 3 = Moderately Motivating

▢ 4 = Motivating

▢ 5 = Very Motivating

7. How useful did you find the smiley/frowning faces and score information shown after correct/incorrect answers?

▢ 1 = Not Useful at all

▢ 2 = Slightly Useful

▢ 3 = Moderately Useful

▢ 4 = Useful

▢ 5 = Very Useful

8. How motivating did you find the security levels (Analyst Candidate, Analyst, Senior Analyst) and rank system in the task?

▢ 1 = Not Motivating at all

▢ 2 = Slightly Motivating

▢ 3 = Moderately Motivating

▢ 4 = Motivating

▢ 5 = Very Motivating

9. Do you think the gamification elements (narrative, progress, score, etc.) made it easier or harder to complete the task?

▢ 1 = Definitely Made it Harder

▢ 2 = Slightly Made it Harder

▢ 3 = No Effect

▢ 4 = Slightly Made it Easier

▢ 5 = Definitely Made it Easier

10. Overall, how satisfied were you with the gamified memory task experience?

▢ 1 = Not Satisfied at all

▢ 2 = Slightly Dissatisfied

▢ 3 = Neither Satisfied nor Dissatisfied

▢ 4 = Satisfied

▢ 5 = Very Satisfied
